# Global Abortion Policies Database: a descriptive analysis of the legal categories of lawful abortion

**DOI:** 10.1186/s12914-018-0183-1

**Published:** 2018-12-20

**Authors:** Antonella F. Lavelanet, Stephanie Schlitt, Brooke Ronald Johnson, Bela Ganatra

**Affiliations:** 1 Department of Reproductive Health and Research and UNDP-UNFPA-UNICEF-WHO-World Bank Special Programme of Research, Development and Research Training in Human Reproduction (HRP), World Health Organization, 20 Avenue Appia, CH-1211, 27 Geneva, Switzerland; 2 UNDP-UNFPA-UNICEF-WHO-World Bank Special Programme of Research, Development and Research Training in Human Reproduction (HRP), World Health Organization, 20 Avenue Appia, CH-1211, 27 Geneva, Switzerland

**Keywords:** Lawful abortion, Abortion legal categories, Abortion laws, Abortion on request, Legal grounds, Indications for abortion

## Abstract

**Background:**

Texts and interpretations on the lawfulness of abortion and associated administrative requirements can be vague and confusing. It can also be difficult for a woman or provider to know exactly where to look for and how to interpret laws on abortion. To increase transparency, the Global Abortion Policies Database (GAPD), launched in 2017, facilitates the strengthening of knowledge and understanding of the complexities and nuances around lawful abortion as explicitly stated in laws and policies.

**Methods:**

We report on data available in the GAPD as of May 2018. We reviewed the content and wording of laws, policies, standards and guidelines, judgments and other official statements for all countries where data is available in the GAPD. We analyzed data for 158 countries, where abortion is lawful on the woman’s request with no requirement for justification and/or for at least one legal ground, including additional indications that are nonequivalent to a single common legal ground. We classified laws on the basis of the explicit wording of the text. The GAPD treats legal categories as the circumstances under which abortion is lawful, that is, allowed or not contrary to law, or explicitly permitted or specified by law.

**Results:**

32% of countries allow or permit abortion at the woman’s request with no requirement for justification. Approximately 82% of countries allow or permit abortion to save the woman’s life. 64% of countries specify health, physical health and/or mental (or psychological) health. 51% allow or permit abortion based on a fetal condition, 46% of countries allow or permit abortion where the pregnancy is the result of rape, and 10% specify an economic or social ground. Laws may also specify several additional indications that are nonequivalent to a single legal ground.

**Conclusions:**

The GAPD reflects details that exist within countries’ laws and highlights the nuance within legal categories of abortion; no assumptions are made as to how laws are interpreted or applied in practice. By examining the text of the law, additional complexities related to the legal categories of abortion become more apparent.

## Background

Abortion is one of the few health procedures that is legally regulated in most countries, but this was not always the case. There were few restrictions on abortion prior to the nineteenth century; women could access abortion prior to quickening, the time at which a woman can feel fetal movement [1]. However, with growing concern related to surgical and medical infection risks, abortion came to be seen as a dangerous and life-threatening surgery, prompting greater regulation, including the inclusion of abortion in penal legislation. In addition to health justifications, restrictions were also based on religious ideology, regulating fertility, fetal protection including for eugenic purposes, and in some cases, desires by physicians to limit competitor practice [1, 2]. These restrictions were progressively incorporated into countries around the world, threatening the lives and eroding the rights of women around the world [3, 4].

In the twentieth century, some countries began to recognize the equal status of women [1], while other countries began to appreciate the dangers of unsafe abortion [3, 5] leading to the liberalization of abortion laws and/or the enactment of new abortion laws [6]. Where abortion is allowed or permitted, three broad categories exist: 1) abortion on request with no requirement for justification; 2) based on common legal grounds and related indications (hereinafter referred to as legal grounds); or 3) based on additional indications that are nonequivalent to a single legal ground but could be interpreted under multiple grounds. Common legal grounds include abortion to save the woman’s life, to preserve the woman’s health, in cases of rape, incest, fetal impairment, and for economic or social reasons [7]. Abortion regulation may occur in legal texts beyond the penal code, including reproductive health acts, general health acts, and medical ethics codes.

Although expanded categories of lawful abortion potentially yield greater access to abortion, the way in which abortion is expressed in legal texts can be vague and confusing. When looking merely at the legal texts, women and providers may find it difficult to know when abortion is lawful and how to interpret information related to legal requirements to ensure compliance with the law. Additionally, abortion may be regulated as a health procedure; abortion may be criminalized in all cases; there may be uncertain prohibition where laws prohibit unlawful abortion but do not specify what constitutes a lawful abortion; or exceptions for permitted abortion access may be specified in the law. Such regulations may exist in a variety of documents including penal codes, ministerial decrees, abortion-specific acts, and court cases to name a few. The expanding range of regulatory documents can sometimes lead to conflicting directives in various sources or even within the same source [7, 8] leading to even greater confusion for women and providers related to the circumstances under which abortion is lawful.

Several databases currently exist which provide information related to country specific abortion laws and may facilitate better understanding of the legal regulation of abortion [7, 9, 10]. These databases often classify countries as falling on a hierarchical spectrum of access to abortion based on the number and type of grounds under which abortion is permitted. To increase transparency, the Global Abortion Policies Database (GAPD) was launched in 2017 [11] and facilitates the strengthening of knowledge by demonstrating the complexities and nuances of legal texts. The GAPD also contains information related to authorization and service-delivery requirements, conscientious objection, penalties, national SRH indicators, and UN Treaty Monitoring Body concluding observations on abortion. The GAPD does not offer information related to the meaning of legal texts or how legal texts are interpreted or applied in society. The meaning of any legal text is informed by its context: the wider set of laws concerning access requirements and women’s reproductive health more generally, and the culture in which these texts are operationalized. However, the GAPD does provide a starting point from which to understand legal categories, including on request with no requirement for justification, legal grounds, and additional nonequivalent indications as set out in national laws.

In this paper, our main objective is to use data extracted from the GAPD, to report on the number of countries that allow or permit abortion within each legal category and describe the complexities and nuances of these laws, which have not been addressed by other databases or have been obscured by more simplistic classification schemes.

## Methods

We use data available in the GAPD as of May 2018.^1^ The GAPD contains data that was extracted onto a policy questionnaire, based on closed questions and a finite set of legal grounds. Unique or complex policy nuances that do not exactly match one of the common legal grounds are separately captured in the GAPD as *other*.^2^ The methodologic details related to the classifying and coding used for the GAPD have been previously described [12]. In this paper, we diverge from the way in which legal grounds are displayed on the GAPD to better describe the complexities related to legal categories of abortion; we do not present data related to additional access requirements.

The GAPD treats legal categories as the circumstances under which abortion is lawful, that is, allowed or not contrary to law, or explicitly permitted or specified by law (legal grounds). We reviewed the content and wording of laws, policies, standards and guidelines, judgments and other official statements (referred to hereinafter as ‘law’ and ‘laws’) for all countries where abortion is lawful on the woman’s request with no requirement for justification and/or for at least one legal ground, including additional indications that are nonequivalent to a single legal ground. Countries where abortion is prohibited in all circumstances (Andorra, Dominican Republic, El Salvador, Gabon, Guinea-Bissau, Haiti, Holy See, Madagascar, Malta, Nicaragua, Palau, Philippines, Republic of Congo, San Marino, Senegal, and Suriname) and countries where laws prohibit unlawful abortion but do not specify lawful abortion (Antigua and Barbuda, Dominica, Gambia, Jamaica, Sierra Leone, Saint Kitts and Nevis, and Tonga) are also excluded.

We only report on data that is available in the GAPD. Countries which have no data available in GAPD include Democratic People’s Republic of Korea, Equatorial Guinea, Honduras, Maldives, Marshall Islands, Micronesia, Niue, and Saint Vincent and the Grenadines. Seven countries (Australia, Bosnia and Herzegovina, Canada, China, Mexico, Nigeria, the United Kingdom of Great Britain and Northern Ireland) that may regulate abortion at the subnational level are not included in the analysis as the GAPD may not have subnational level data or the data may vary significantly across the jurisdictions. Thus, we analyzed data for 158 countries.

The coding and classification of laws is based on the explicit text of the law. We do not make assumptions about the interpretation of laws. Each ground is treated independently; countries where abortion is permitted on request with no requirement for justification are not coded in the database as countries that permit any other legal ground unless those grounds are explicitly stated. The information in the database is limited by accessibility of source documentation and the ability to translate source documents.

## Results

### On request with no requirement for justification

Abortion at the woman’s request with no requirement for justification is allowed or permitted in 50 countries (32% = 50/158); just over half of these are in Europe (54% = 27/50). In Asia, there are 14 countries where abortion on request is lawful, followed by six in Africa, three in Latin America and the Caribbean, and one in North America; there are no countries in Oceania where abortion is lawful on the woman’s request with no requirement for justification. All but one country (Viet Nam) impose gestational age limits on women accessing abortion on request.^3^ In all other countries, abortion on request is typically available up to 12 weeks of gestation; the range is 8 to 24 weeks.

### Legal grounds and related indications

Where abortion is not available on request or once the gestational limit associated with a woman’s request has been reached, abortion may be lawful based on legal grounds or related indications.

#### Life threat

Approximately 82% (129/158) of countries allow or permit abortion to save the woman’s ‘life’ (See Table 1). The threat to life is described in various ways.

**Table 1:** Regional data: Life ground and associated gestational limits

	Africa	Asia	Europe	Latin America and Caribbean	North America	Oceania	Total
Total number of countries included in analysis	45	43	38	21	1	10	158
Total number of countries with life ground	36	32	32	19	1	9	129
Gestational limit < 12 weeks	1	0	0	0	n/a*	0	1
Gestational limit 12 to 18 weeks	2	2	0	0	n/a*	0	4
Gestational limit 19 to 24 weeks	0	5	8	4	n/a*	1	18
Gestational limit > 24 weeks	3	0	1	0	n/a*	0	4
‘viability’	3	0	1	0	n/a*	0	4
Total with gestational limits	9	7	10	4	n/a*	1	31

*North America = United States of America; Gestational limits vary by jurisdiction

Some laws reference the threats/risks the pregnant woman confronts as circumstances in which:

*‘continuation of pregnancy endangers the life.’*

Others qualify the level of the threat/risk the pregnant woman confronts when:

*‘there is a substantial threat to the woman’s life in continuing the pregnancy.’*

Yet others compare the risks the woman confronts:

*‘if the continuance of the pregnancy would involve a risk to the life of the woman greater than if the pregnancy were terminated”* or where “*abortion is the only way to save the woman’s life.*’

Seven of the 129 countries^4^ (5%) utilize a medical or surgical operations clause to permit abortion to save the woman’s life, which exempts from criminal responsibility those who perform ‘in good faith and with reasonable care and skill a surgical operation upon an unborn child for the preservation of the mother’s life, if the performance of the operation is reasonable, having regard to the patient’s state at the time and to all the circumstances of the case.’

In 24 of the 129 countries (19%) where abortion is lawful based on a life threat,^5^ this indication is the only permissible circumstance in which a woman may lawfully obtain an abortion. Most countries do not impose gestational age limits related to the life ground; however, gestational limits are present in 31 countries across the regions (See Table 1).

#### Health threat

**Fig. 1:**
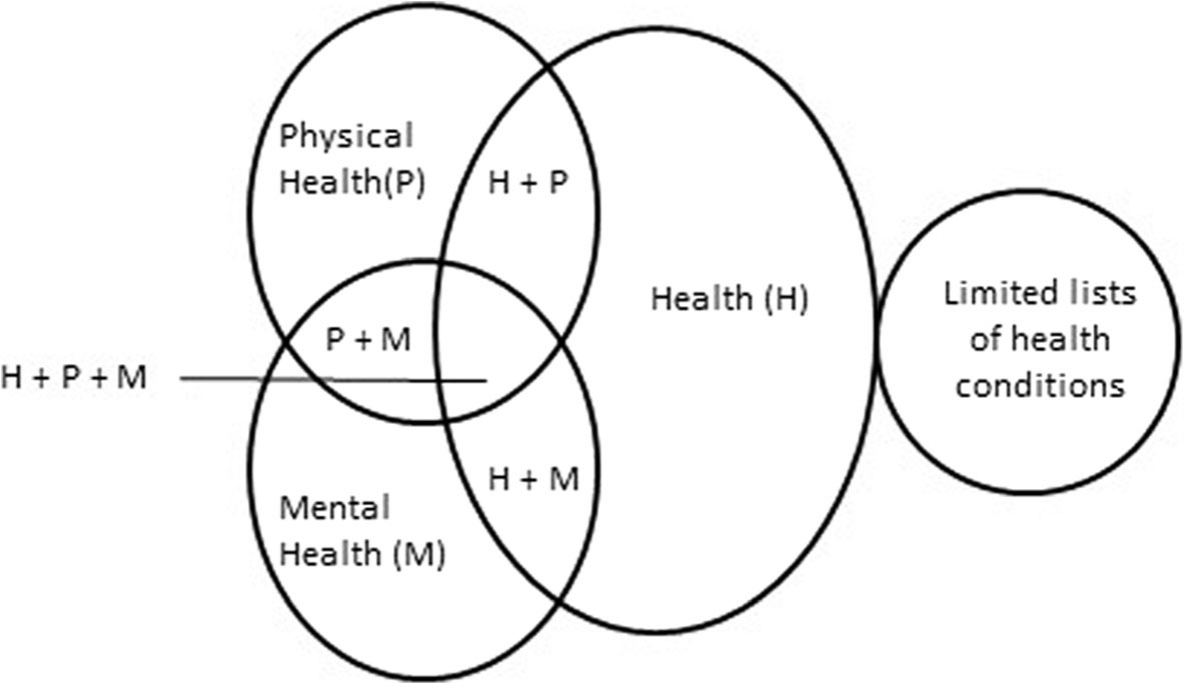
Relationship between health, physical health and/or mental health ground and related indications

The laws of most countries with a health-related ground refer to one or a combination of the following terms: ‘health’, ‘physical health’ and/or ‘mental (or psychological) health.’ Some laws specify limited lists of health conditions (See Fig. 1).

Of 158 countries analyzed, 101 (64%) specify health in some form. Health alone is specified in 49 (31% = 49/158) countries; 36 (23% = 36/158) additional countries provide greater detail in their laws, specifying the lawfulness of abortion based on both ‘physical health’ and ‘mental health.’ In 10 countries,^6^ laws specify ‘health,’ ‘physical health’ and ‘mental health.’ In Japan and Mongolia, ‘health’ and ‘physical’ health are specified, while in Finland and Iraq, ‘health’ and ‘mental health’ are specified. In Monaco and Zimbabwe, abortion may only be lawful based on a ‘physical health’ ground.

In addition to specifying ‘health’, ‘physical health’ and/or ‘mental (or psychological) health,’ 9 countries^7^ narrow the lawfulness of abortion to certain specified health conditions, including HIV infection, severe depression or where a woman’s psychological equilibrium may be compromised by continuation of the pregnancy.

Most countries do not impose a gestational limit for any health indication, but in the 38 countries (38% = 38/101) where the law specifies an associated gestational limit, most fall between 19 and 24 weeks (See Table 2).

**Table 2:** Regional data: Health, physical health and/or mental health ground and associated gestational limits

	Africa	Asia	Europe	Latin America and Caribbean	North America	Oceania	Total
Total number of countries included in analysis	45	43	38	21	1	10	158
Total number of countries with health, physical health and/or mental health ground	31	20	33	13	1	3	101
Gestational limit < 12 weeks	0	0	0	0	n/a	0	0
Gestational limit 12 to 18 weeks	4	3	1	0	n/a	0	8
Gestational limit 19 to 24 weeks	2	6	10	3	n/a	1	22
Gestational limit > 24 weeks	3	0	2	0	n/a	0	5
‘viability’	1	0	2	0	n/a	0	3
Total with gestational limits	10	9	15	3	n/a*	1	38

*North America = United States of America; Gestational limits vary by jurisdiction

#### Limited lists of health conditions

Six countries (Azerbaijan, Georgia, Kyrgyzstan, Russian Federation, Tajikistan, and Turkey) have limited lists of specific health conditions; several types of diseases may be included on such lists. In one country, for example, the category “infectious and parasitic diseases” includes all active forms of tuberculosis, severe viral hepatitis, syphilis, Acquired Immunodeficiency Syndrome and rubella. The category “mental disorders” includes chronic alcoholism with personality change, transient psychotic conditions resulting from organic diseases, drug addiction and substance abuse, and mental retardation.

#### Fetal conditions

**Fig. 2:**
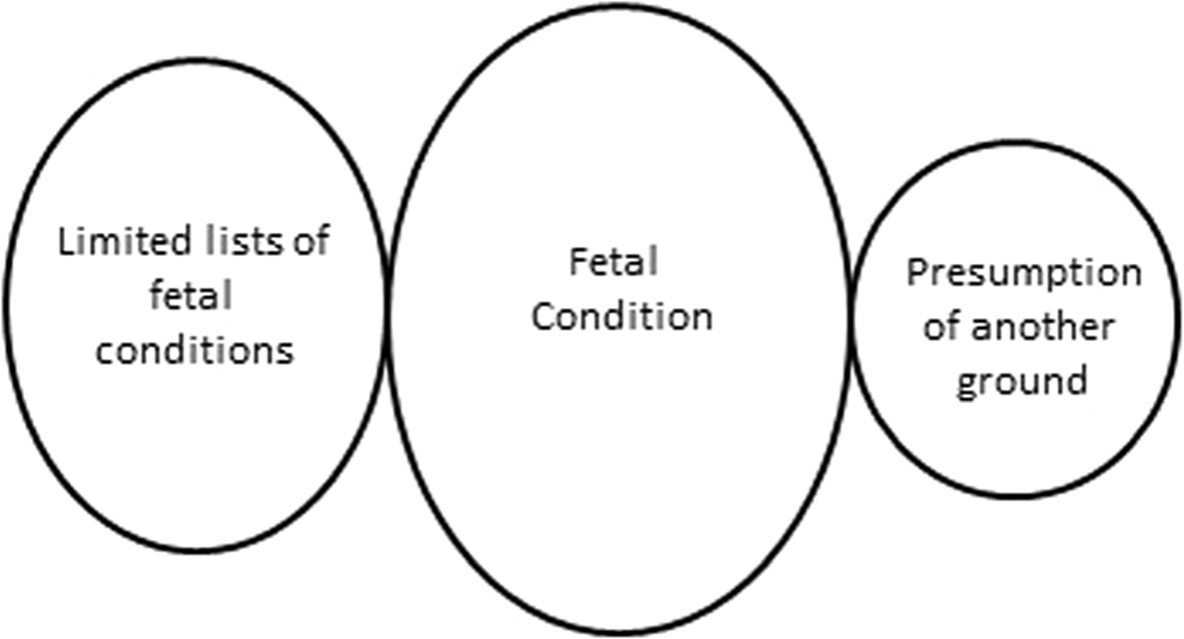
Relationship between a ground based on fetal condition and related indications

Laws allow or permit access to abortion based on fetal conditions; in some cases, countries provide a limited list of conditions or specify a single fetal condition for which abortion is lawful. (See Fig. 2).

In 80 of the 158 (51%) countries analyzed, abortion is allowed or permitted based on a fetal condition, with no restriction as to the type of fetal condition (See Table 3). In 35 of these 80 countries, gestational limits restrict a woman’s access to abortion based on a fetal condition. These gestational ages range from 8 to 35 weeks; the median is 22 weeks.

**Table 3:** Regional data: Ground based on fetal condition and associated gestational limits

	Africa	Asia	Europe	Latin America and Caribbean	North America	Oceania	Total
Total number of countries included in analysis	45	43	38	21	1	10	158
Total number of countries with ground for fetal condition	26	19	28	5	n/a*	2	80
Gestational limit < 12 weeks	1	0	0	0	n/a	0	1
Gestational limit 12 to 18 weeks	3	4	1	1	n/a	0	9
Gestational limit 19 to 24 weeks	3	4	9	1	n/a	1	18
Gestational limit > 24 weeks	3	0	1	1	n/a	0	5
‘viability’	1	0	1	0	n/a	0	2
Total with gestational limits	11	8	12	3	n/a	1	35

*North America = United States of America

#### Limited lists of or single specified fetal condition

In six countries, an abortion is lawful if the fetus has a congenital or hereditary disease (Bulgaria and Lithuania), or where the fetus’s condition is lethal (Bolivia and Colombia) or ‘incompatible with extrauterine life’ (Chile and Uruguay). In Brazil, an anencephalic fetus is the only lawful fetal condition.

#### Fetal condition - presumption of another ground

In Thailand, if the woman suffers ‘severe stress’ due to the finding that the fetus is afflicted with a ‘severe disability, or has or has a high risk of having severe genetic disease,’ abortion is lawful under the mental health ground. A medical practitioner, other than the one who will perform the termination of pregnancy must authorize the abortion based on this ground in writing.

#### Rape

**Fig. 3:**
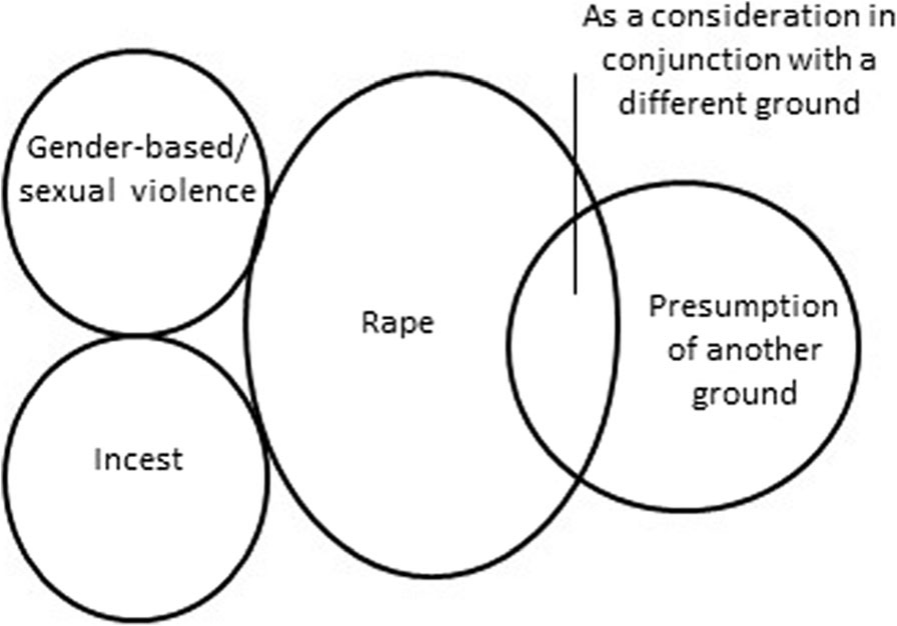
Relationship between rape ground and related indications

Many countries allow or permit abortion in cases where the pregnancy is the result of rape or gender-based/sexual violence; however, laws vary in how this ground is defined (See Fig. 3).

Abortion is lawful in 72 of the 158 countries analyzed (46%) if pregnancy is the result of ‘rape.’ In 61% (44/72) of countries where rape is a permitted legal ground, an accompanying gestational limit is imposed. The range between the lowest and highest limits varies across regions (See Table 4).

**Table 4:** Regional data: Rape ground and associated gestational limits

	Africa	Asia	Europe	Latin America and Caribbean	North America	Oceania	Total
Total number of countries included in analysis	45	43	38	21	1	10	158
Total number of countries with rape indication	25	15	20	11	n/a*	1	72
Gestational limit < 12 weeks	2	1	0	1	n/a	0	4
Gestational limit 12 to 18 weeks	5	1	5	4	n/a	0	15
Gestational limit 19 to 24 weeks	3	6	9	2	n/a	1	21
Gestational limit > 24 weeks	3	0	1	0	n/a	0	4
‘viability’	0	0	0	0	n/a	0	0
Total with gestational limits	13	8	15	7	n/a	1	44

*North America = United States of America

#### Rape - presumption of another ground

In Barbados and India, abortion is lawful under the mental health ground where pregnancy results from rape. In both countries there is a presumption that pregnancy resulting from rape is or can be injurious to the woman’s mental health, without the need for a health professional’s assessment.

#### Rape - as a consideration in conjunction with a different ground

One country, New Zealand, specifically states in its law that rape is not in and of itself a legal ground but may be considered if there are reasonable grounds for believing that the pregnancy is the result of sexual violation, where continuation of the pregnancy would result in serious danger to the woman or girl’s life, physical or mental health.

#### Gender-based/sexual violence

In 14 countries where abortion is permitted on the ground of rape, abortion is also allowed or permitted if the pregnancy is the result of another specific act of sexual violence including human trafficking, forced marriage, sexual assault, or unwanted implantation of a fertilized ovum.

In the laws of six countries, rape is not an explicit indication for abortion, however, similar indications exist. The laws in four countries (Angola, Bulgaria, Italy and Portugal) permit consideration of the circumstances in which the pregnancy occurred, such as if the pregnancy was the “result of a crime against freedom and sexual self-determination” or resulted “from an act of violence.” In Zambia and Bolivia, specific acts of gender-based violence, such as defilement or forced marriage are included in the law.

#### Incest

Of 45/72 (63%) countries that have a rape ground, abortion is also lawful if the pregnancy is the result of ‘incest’. Two countries (Bulgaria and New Zealand) do not explicitly specify a rape ground in their laws but do allow or permit abortion where the pregnancy is the result of incest. Gestational limits restrict a woman’s access to abortion based on incest in 26 of the 45 countries where abortion is lawful. These gestational ages range from 8 to 28 weeks; the median is 20 weeks.

#### Intellectual or cognitive disability

In 20 of the 158 countries, intellectual or cognitive disability of the woman is specified as a legal ground. In 13 of these 20 countries, gestational limits restrict the application of this indication. The range is between 12 and 28 weeks, the median is 21 weeks.

#### Economic or social ground

**Fig. 4:**
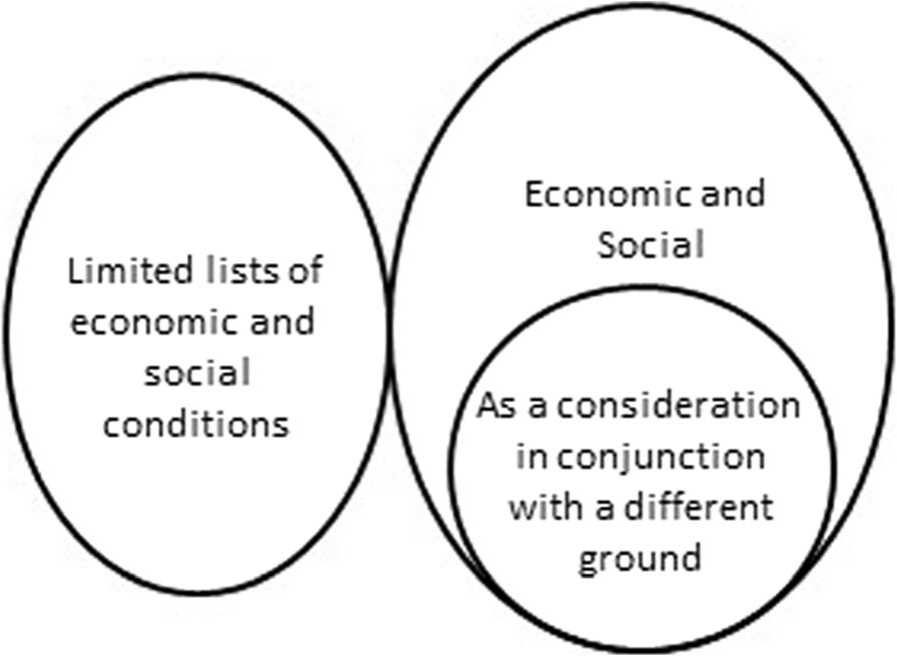
Relationship between economic or social ground and related indications

Economic and/or social grounds are specified in laws either as an independent ground or as a consideration in conjunction with a different ground. Alternatively, some countries’ laws have limited lists of or a single specific economic or social condition (See Fig. 4).

Of 158 countries analyzed, 16 countries (10%) allow or permit abortion based on an economic or social ground. In 13 of the 16 countries where abortion is permitted on an economic and social ground, gestational limits restrict the application of this indication. The range is between 12 and 22 weeks, the median is 21 weeks.

#### Economic or social ground -as a consideration in conjunction with a different ground

Six countries permit consideration of economic and social reasons in conjunction with another ground. In Barbados, Belize, and Zambia, a pregnant woman’s actual or foreseeable social environment may be considered in determining whether a risk to her life or health exists. Similarly, a woman’s living conditions or economic circumstances may be taken into account in Germany and Guyana where abortion is considered justified to avert injury to her health. In The former Yugoslav Republic of Macedonia, an abortion is lawful if a woman has seriously deteriorated marital and family relations or a difficult housing condition and these circumstances may be detrimental to her health.

#### Limited lists of or single specified economic and social conditions

In 16 countries, specific social indications or a limited list of social indications are specified within their laws. For example, in Israel, abortion is lawful where the pregnancy is the result of extramarital relations. In Guyana and Slovakia, abortion is permitted in cases of contraceptive failure. In Kazakhstan, the law includes a list of social circumstances, such as the death of a woman’s husband, the woman and her husband are recognized as officially unemployed, refugee status for the woman, and if the woman has four or more children, to name a few.

### Non-equivalent indications

Abortion may also be lawful based on indications that are not equivalent to a single legal ground.

#### Claim of distress

In four countries, the law allows or permits abortion in the first 12 weeks of pregnancy to women who suffer from distress or similar impact from continuation of the pregnancy. In the Netherlands, a woman’s request for abortion must be based on her opinion that she is in an emergency situation which can only be alleviated by an abortion. In Switzerland, abortion is lawful if a woman provides a written request claiming that she is in distress. In Belgium and Hungary, the woman must be distressed or in a crisis situation, as assessed by her attending doctor.

#### Age qualification

In 22 countries, abortion is lawful for minors, or those below or above a specified age. In these countries, abortion is typically permitted for girls between 13 and 18 years of age, and women over 40 years. In 14 of the 22 countries, the law allows or permits abortion at one end of this age spectrum, either for those before 18 or after 40 years of age. In 6 countries, minority is accompanied by additional stipulations. For example, in Liberia, a girl under 16 is entitled to an abortion where the pregnancy is the result of illicit intercourse. In Denmark and Ethiopia, a minor whose immaturity renders her unfit to raise a child may have an abortion. In Liechtenstein, a girl under 15 is entitled to an abortion, if she is not married to the person who impregnated her at the time of conception or afterwards. In Benin and Central African Republic, where the pregnancy would constitute a handicap for the minor’s development or lead to a state of grave distress, abortion is lawful.

#### Various therapeutic indications

In 17 countries, the law allows or permits abortion in circumstances that may be categorized as potentially falling under several common grounds, including life, health, fetal condition, economic and social reasons, and rape. These countries’ laws allow or permit abortion where such procedures are for a “‘therapeutic purpose’ or ‘proven medical necessity,”’ to ‘avert the danger of serious harm to physical integrity’ or to prevent ‘serious and irreversible harm to the body.’ Some of these laws allow or permit abortion where a spouse suffers from a mental disease.

Two additional countries (Bahamas and Grenada) have a surgical operations clause but make no reference to preservation of the ‘mother’s’ life, while one country (Mozambique) permits health committees to examine cases not stipulated in the law on a case-by-case basis to protect pregnant women’s’ sexual and reproductive rights.

#### Menstrual regulation

In Bangladesh, ‘menstrual regulation’ is medically or surgically available to women as a method of uterine evacuation used to regulate the menstrual cycle when menstruation has been absent for a short duration.

## Discussion

While the GAPD does not provide information on how laws are interpreted or applied in practice, this analysis demonstrates that there are wide variations in how countries specify legal categories, including abortion on a woman’s request with no requirement for justification, legal grounds, and additional, but non-equivalent indications. Unpacking each category and revealing the nuances that exist within legal texts acts a starting point to the discourse around when abortion is allowed or permitted.

### Determining what is included within a legal category

The circumstances under which abortion is lawful may be unclear to women and service providers attempting to navigate vague or complex laws**.** The World Health Organization (WHO) describes health as “a state of complete physical, mental and social well-being and not merely the absence of disease or infirmity” [13]. While all WHO Member States accept this definition of health, many countries’ laws do not refer to either the WHO definition or reference explicitly all the component parts of health. The results demonstrate that sometimes health is specified in a variety of ways in legal texts. Where laws contain a specific list of health indications for which an abortion can be performed, questions may arise as to whether service providers will interpret these lists restrictively or whether they will consider them as illustrations, which do not preclude clinical judgment [14]. Where mental health is not specified, it may not necessarily mean that women with mental health conditions are now lawfully entitled to abortion, given that service providers exercise sole discretion as to whether these conditions will be considered under a more broadly framed ‘health’ indication. Similarly, in cases such as New Zealand, where rape may be considered if the woman is faced with a serious danger in terms of a threat to her life or her physical or mental health, questions arise as to how that effect is assessed.

However, there may be value in the law being vague as it relates to health and other grounds, as access may be available more broadly, in line with the WHO definition. Similarly, countries’ laws that contain vague language, such as ‘therapeutic purposes’, ‘very serious medical reasons’, or ‘necessary treatment to the woman’ may permit health-care providers to apply these indications consistent with their obligation to the health and well-being of their patients. Thus, these indications may apply when there is a threat to the woman’s life or health, in cases where a fetal condition is present, or where a woman faces economic or social circumstances requiring necessary treatment.

Physicians may also apply such grounds against the knowledge that women may seek clandestine abortion, which depending on the context, can pose risks to life and health [14]. In one country (Bangladesh), despite a restrictive penal code, which offers only a life ground for abortion, menstrual regulation is a lawful way to “to reduce the incidence of unwanted pregnancies and unsafe abortions” [15]. Specifically, menstrual regulation is available “as a backup family planning method” to women with a last menstrual period of 10 weeks or less who may be “at risk of pregnancy, whether or not [they are] actually pregnant” [15]. Providers may also appreciate the risks associated with a continued pregnancy, including the fact that 75% of global maternal deaths are a result of direct obstetric causes [16], or that mortality associated with childbirth is approximately 14 times higher than that of abortion [17].

However, without specified legal categories or clear language, and where severe penalties may exist, health-care providers may interpret legal grounds narrowly, restricting access to safe abortion beyond what the law requires. For example, according to a study in Argentina, interpretation related to the scope of the health ground, as well as whether the rape ground applies to all women or only those with mental disabilities, has hindered access to abortion [18]. Even where a ground is explicitly stated in the law and supported by providers, this same study reveals that only 50% of providers are willing to perform an abortion [18]. These interpretations may be motivated by culture and gender stereotypes [18]. While almost all countries allow or permit abortion on the basis of some life-related ground (*N* = 133), variations in interpretation or lack of appreciation of the severity of the risk can have devastating consequences [19].

Additionally, fear of liability may lead health-care providers to limit access well before a gestational limit has been reached for a permitted legal ground [20]. Interpretation may also impact available methods; for example, service providers may feel they cannot provide medical abortions in countries where the only legal basis for abortion is a medical or surgical operations clause. Thus, greater concerns about abortion access and safety arise when there is lack of clarity related to the law, as providers must balance the risk of potential criminal liability or other self-interests against the needs and desires of the woman.

The legal categories for abortion cannot be neatly packaged into discrete classes based on common legal grounds. It is only by examining the text of the law that nuances are exposed. Subsuming these specific circumstances under common legal grounds provides a false sense of certainty about the legal status and availability of abortion services within a country. For example, in Belgium, Hungary, Netherlands, and Switzerland, countries that have been previously classified as permitting abortion on request, are found on review of the text to permit abortion within the context of a claim of distress where a written or verbal statement is required by the woman describing her situation as one of crisis or emergency or distress.

### Legitimizing or delegitimizing women’s claims to abortion

Laws that specify individual legal grounds reflect the perceived legitimacy of some of the reasons women may have for wanting an abortion. Our analysis demonstrates that in most countries’ laws, abortion based on the legal ground of life threat is the most common, followed by health threat, fetal condition and rape, suggesting a hierarchy in the acceptability of women’s reasons. It could be argued that entitlement to abortion is based on a cumulative effect – the more grounds that exist, the greater the likelihood that women in different circumstances may qualify under one of these grounds. However, this raises questions related to fairness and equity regarding why countries single out specific conditions for entitlement to abortion, especially when women more often seek abortion based on socio-economic issues, age, health, family life, and marital status [21], rather than based on a life threat or rape ground.

This issue is further compounded by associated gestational limits; the wide variation in gestational limits demonstrates that they are not based on evidence. In the case of a fetal impairment indication, for instance, it may be difficult for a woman to comply with a gestational limit of 8 weeks when this time limit is several weeks before usual diagnostic tests are undertaken. Gestational limits narrow a woman’s options as the pregnancy progresses making legal grounds with higher gestational limits appear as more significant than those with lower limits.

Moreover, laws that impose time limits on the length of pregnancy for which abortion can be performed can force some women to seek clandestine abortion or to seek services in other countries, which is costly, delays access (thus increasing health risk) and creates social inequities [22]. It is for this reason that reducing unsafe abortion and abortion-related morbidity and mortality are less related to the total aggregate of grounds available and more related to access based on broad socioeconomic grounds or at the woman’s request [21].

This paper focuses on only one aspect of legal abortion; access must be considered within the broader context of sexual and reproductive healthcare. For example, additional barriers may be linked to legal categories and are often inscribed in the law; such barriers include mandatory waiting periods, requirements for third-party authorizations, conscientious objection, and reporting requirements in cases of rape. Laws related to contraception, financing of abortion, and access to medical information also impact how laws and policies are translated into practice. Additionally, national laws exist within a greater international context. The GAPD includes all UN Treaty Monitoring Body concluding observations and Special Procedures reports that have addressed abortion since the year 2000; human rights and UN treaty bodies have reiterated state’s obligations in terms of regulation of abortion and that the “right to sexual and reproductive health is an integral part of the right to health” [23].

## Conclusions

The GAPD aims to increase transparency of information and accountability of countries for the protection of individuals’ health and human rights in the context of abortion. The database expands on existing knowledge related to the legal categories of abortion by capturing unique or complex policy nuances, a starting point by which to better consider legal entitlements to abortion.

This paper highlights the wide variation that exists in legal texts across countries related to the legal categories of abortion demonstrating several indications that have previously been obscured behind more simplistic classification schemes. Illuminating the complexities that exist reveals additional burdens on women and health-care providers to interpret legal categories related to abortion. Moreover, women seek abortion services based on one or more reasons which do not neatly fit into distinct legal classifications, and providers are relied upon to determine a woman’s eligibility based on their interpretation of these laws, creating an illusion of transparency that does not necessarily reflect the actual scope and potential limits of the law. With so much variance in legal texts, questions arise as to how women and healthcare workers appreciate these nuances both within and among different legal categories. Further research is needed to investigate the interpretation and implementation of these laws in practice, including how abortion legal categories co-exist among other laws related to reproductive health and how they are applied across various social, cultural, political, and economic contexts.

## Abbreviations

GAPD: Global Abortion Policies Database
WHO: World Health Organization

## Footnotes

1 Information in the GAPD changes as new sources are received and verified.

2 ‘Other’ includes countries with caveats, stipulations or countries where additional qualifications linked to a woman’s request are required; these countries are not represented as having abortion on request in the GAPD. Results for these countries are presented as an access ground based on a specific legal indication.

3 In Tajikistan, an order of the Minister of Health containing National Standards for safe abortion and post abortion care exist and may contain information related to gestational limits, but is not reflected here as this source could not be translated.

4 Africa: Malawi and Uganda; Oceania: Kiribati, Nauru, Papua New Guinea, Solomon Islands, and Tuvalu.

5 Africa: Cote d’Ivoire, Democratic Republic of the Congo, Libya, Malawi, South Sudan, Uganda; Asia: Afghanistan, Bahrain, Brunei Darussalam, Lebanon, Myanmar, Oman, Sri Lanka, Syrian Arab Republic and Yemen; Europe: Ireland; Latin America and Caribbean: Guatemala, Paraguay, and Venezuela; Oceania: Cook Islands, Kiribati, Nauru, Papua New Guinea, and Solomon Islands.

6 Argentina, Bolivia, Chad, Columbia, Ecuador, Hungary, Iceland, Thailand, Trinidad and Tobago, and Uruguay.

7 Bulgaria, Cuba, Czech Republic, Guyana, Moldova, Mozambique, Timor Leste, Tunisia, and Uzbekistan.
